# Optimization of Pulse-Field Gel Electrophoresis for Subtyping of *Klebsiella pneumoniae*

**DOI:** 10.3390/ijerph10072720

**Published:** 2013-07-01

**Authors:** Hui Han, Haijian Zhou, Haishan Li, Yuan Gao, Zhi Lu, Kongxin Hu, Baoliang Xu

**Affiliations:** 1 Chinese Academy of Inspection and Quarantine, Beijing 100123, China; E-Mails: hanhui2002@163.com (H.H.); haishanli@163.com (H.L.); kongxinhu@sina.com (K.H.); 2 National Institute for Communicable Disease Control and Prevention, and State Key Laboratory for Infectious Disease Prevention and Control, Chinese Center for Disease Control and Prevention, Beijing 102206, China; E-Mail: gaoyuan7921@yahoo.com.cn; 3 Gaoling Center for Diseases Control and Prevention, Xi’an 710200, China; E-Mail: xiaolu2041@163.com

**Keywords:** *Klebsiella pneumoniae*, pulsed-field gel electrophoresis, molecular subtyping

## Abstract

A total of 110 strains of *Klebsiella pneumoniae* were used to optimize pulsed-field gel electrophoresis (PFGE) for subtyping of *K. pneumoniae*. For optimization of electrophoresis parameters (EPs) of *Xba*I-PFGE, 11 isolates were analyzed with *Xba*I digestion using three EPs. The EP of a switch time of 6 to 36 s for 18.5 h gave clearest patterns and was declared the optimal EP for *Xba*I PFGE of *K. pneumoniae*. By software analysis and pilot study, *Avr*II was chosen as another PFGE enzyme. Both *Xba*I- and *Avr*II-PFGE gave *D*-values higher than 0.99 for 69 *K. pneumoniae* isolated from different sources. Our results also showed good typeability, reproducibility of both *Xba*I- and *Avr*II-PFGE for *K. pneumoniae* subtyping. Furthermore, the established PFGE method also had good discriminatory power to distinguish outbreak *K. pneumoniae* strains and a high degree of consistency with multilocus sequence typing method. A rapid PFGE protocol was established here, which could be used for genotyping and other researches of *K. pneumoniae*.

## 1. Introduction

*Klebsiella pneumoniae* is an opportunistic pathogen that usually causes hospital- and community-acquired bacterial infections in humans. Invasive *K. pneumoniae* has been reported to be an emerging infectious disease causing pyogenic liver abscesses and complications, such as meningitis or endophthalmitis [1,2,3]. The emergence and rapid spread of drug-resistant *K. pneumoniae* isolates is becoming a serious antibiotic management problem and causing great concern worldwide [4,5].

A variety of subtyping techniques have been used to identify and characterize *K. pneumoniae* strains, including ribotyping [6], amplified fragment length polymorphism (AFLP) analysis [7], multilocus sequence typing (MLST) analysis [8] and pulsed-field gel electrophoresis (PFGE) [9,10]. Among them PFGE is the mostly used one. Standardized PFGE protocols had been established for pathogens such as *Salmonella* spp., *Shigella* spp., *Escherichia coli* O157:H7, *Vibrio cholerae* and *Vibrio parahaemolyticus* [11,12,13]. The use of standardized PFGE protocols allows for rapid comparison of DNA fingerprints between different laboratories to enhance disease surveillance. However, laboratories that use PFGE to analyze *K. pneumoniae* cannot compare their results because the protocols differ from each other in critical parameters, especially the restriction enzymes and electrophoresis conditions [14,15,16], so there is a need for an optimized PFGE protocol for *K. pneumoniae* subtyping which can readily be implemented in different laboratories for data interpretation.

The present study was performed to develop an optimized PFGE protocol for subtyping of *K. pneumoniae* in which we chose the standard protocol used in plug preparation for other bacterial species. We chose two enzymes and a set of electrophoresis parameters (EPs) to obtain good discriminatory power. The study was also designed to assess the typeability, reproducibility and concordance with other molecular typing method of PFGE in *K. pneumoniae*.

## 2. Materials and Methods

### 2.1. Bacterial Strains and Culture Conditions

One hundred and ten strains of *K. pneumoniae* were used in this study. Isolates were collected from three sources: one hospital in Beijng city in 2012, one hospital in Chengdu city in 2005, and one health service center in Shenyang city during 2010–2011. The strains were stored at −70 °C in brain heart broth with 20% sterile glycerol in our lab. All strains were routinely cultured on Mueller-Hinton (MH) agar plates, and typical colonies were picked up, identified by biochemical tests using the API^®^-20E test kits (bioMérieux, Lyon, France). The bacteria were grown at 37 °C for 18–24 h for preparation of bacterial suspension and DNA extraction.

### 2.2. PFGE Protocol

The PFGE protocol used was based on the PulseNet 1-day standardized PFGE protocol for *Escherichia coli* O157:H7, *Salmonella*, and *Shigella* [13]. The cell suspension, placed in a polystyrene tube (Falcon; 12 by 75 mm), was adjusted to a density of 3.8–4.2 units using a bioMérieux DENSIMAT. A 400-μL aliquot of the adjusted cell suspension was transferred to a 1.5-mL centrifuge tube and 20 μL of proteinase K (20 mg/mL stock; Invitrogen, Carlsbad, CA, USA) was added and mixed gently with the pipette tip. Plugs were made by adding an equal volume (400 μL) of molten 1.0% SeaKem Gold (SKG, Cambrex, Rockland, MD, USA) agarose to each cell suspension, gently mixing by pipetting up and down several times and the mixture was immediately dispensed into the wells of a reusable plug mold (Bio-Rad Laboratories, Hercules, CA, USA). The agarose plugs were allowed to solidify for 10 min at room temperature. The plugs were transferred into 50-mL polypropylene tubes containing 5 mL of cell lysis buffer (50 mM Tris, 50 mM EDTA (pH 8.0), 1% sarcosine, and 0.5 mg of proteinase K/mL). Lysis of cells in the plugs was performed for 2 h at 54 °C in a water bath with constant agitation (160 rpm). The cell lysis buffer was removed and the plugs were washed two times with 15 mL of sterile ultrapure water and four times with 15 mL of TE buffer (10 mM Tris:1 mM EDTA (pH 8.0)). Each washing step was performed at 50 °C for 15 min with constant agitation. Following the final wash, the TE was removed and replaced with 5 mL of fresh TE buffer at room temperature. Slices of *K. pneumoniae* plugs were digested in a volume of 200 μL with corresponding amount per slice enzymes (New England Biolabs, Ipswich, MA, USA) for 4 h at 37 °C. The electrophoresis was run on the CHEF-DRIII system (Bio-Rad Laboratories). Images were captured on the Gel Doc 2000 system (Bio-Rad) and converted to TIFF for computer analysis. The plugs of H9812 were prepared and digested along with test strains. Slices of H9812 were digested with 40 U/slice *Xba*I (TaKaRa Bio, Dalian, China). All electrophoreses were run with a voltage gradient of 6 v/cm, an included angle of 120° and a liner ramp. *Salmonella* serotype Braenderup H9812 was used as a DNA size marker, as recommended by PulseNet [17].

### 2.3. Computer Analysis of PFGE Patterns

The PFGE patterns were analyzed using BioNumerics software package (version 5.10, Applied Maths, Inc., Austin, TX, USA). Similarity analysis was performed by Dice coefficients (S_D_) (Dice 1945) with customized tolerance of 1.5%. *S_D_* is calculated as *S_D_* = [2(*n_xy_*)]/(*n_x_* + *n_y_*), where *n_xy_* is the number of bands common to isolates *X* and *Y*, *n_x_* is the total number of bands for isolate *X*, and *n_y_* is the total number of bands for isolate *Y*. Clustering was created using unweighted pair group with arithmetic averages (UPGMA). The fragments smaller than 20.5 kbp were not analyzed.

### 2.4. Enzyme Selection

The preliminary enzymes were selected using DNASTAR 5.01 software (DNASTAR, Inc., Madison, WI, USA) based on the complete genome sequence of *K. pneumoniae* subsp. *pneumoniae* MGH 78578 (GenBank accession No. NC_009648; CP000647). The primary enzymes were then selected. A pilot test using four strains was conducted for further evaluation and the candidate enzymes for *K. pneumoniae* PFGE were selected based on the distribution of the bands. The candidate enzymes were further evaluated for use in PFGE of *K. pneumoniae*.

### 2.5. Optimization of Electrophoresis Parameters (EP) for Optimal Enzyme Digestion

Eleven isolates were analyzed with each candidate enzymes digest using three EPs: EP-a, EP-b and EP-c (Table 1). EP-a was recommended by the CHEF Mapper equipment manufacturer. EP-b and -c, were fine-tuned based on EP-a to provide the best possible resolution. Simpson diversity index (*D*-value) was used to compare the discriminatory power under each parameter [18]. *D*-value was given by the equation *D* = 1 − {∑ [*n_j _*(*n_j_* − 1)]}/[*N* (*N* − 1)], where *n_j_* is the number of strains belonging to the *j*th type and *N* is the number of strains in the population. The similarity coefficients of every two PFGE patterns were compared. Two-tailed probability was calculated using the Friedman test by SPSS 11.5 for multi-group comparisons. The Friedman test is a non-parametric test for analyzing randomized complete block designs, testing the null hypothesis that the treatments have identical effects. If significance was achieved among groups, The Friedman test was performed for two-group comparisons with the adjusted significance level of 0.007. An EP with higher discriminatory power can distinguish patterns better, thus yielding a smaller similarity coefficient. Accordingly, the EP with higher *D*-value and minimal similarity coefficients was considered optimal for distinguishing strains and was used as the standard to evaluate the discriminatory power of the enzymes selected.

**Table 1:** Features of four electrophoretic parameters with *Xba*I digestion.

Electrophoresis parameters	Switch time (s)	Total run time (h)
EP-a	6–20	18
EP-b	6–36	18.5
EP-c	4–40	19

### 2.6. Multilocus Sequence Typing

Multilocus sequence typing (MLST) with seven genes (*gapA*, *infB*, *mdh*, *pgi*, *phoE*, *rpoB* and *tonB*) was performed on isolates according to the protocol described on the *K. pneumoniae* MLST database [19]. Alleles and sequence types (STs) were assigned by using the *K. pneumoniae* MLST database [19].

## 3. Results

### 3.1. Optimization of Electrophoretic Parameters

**Figure 1:**
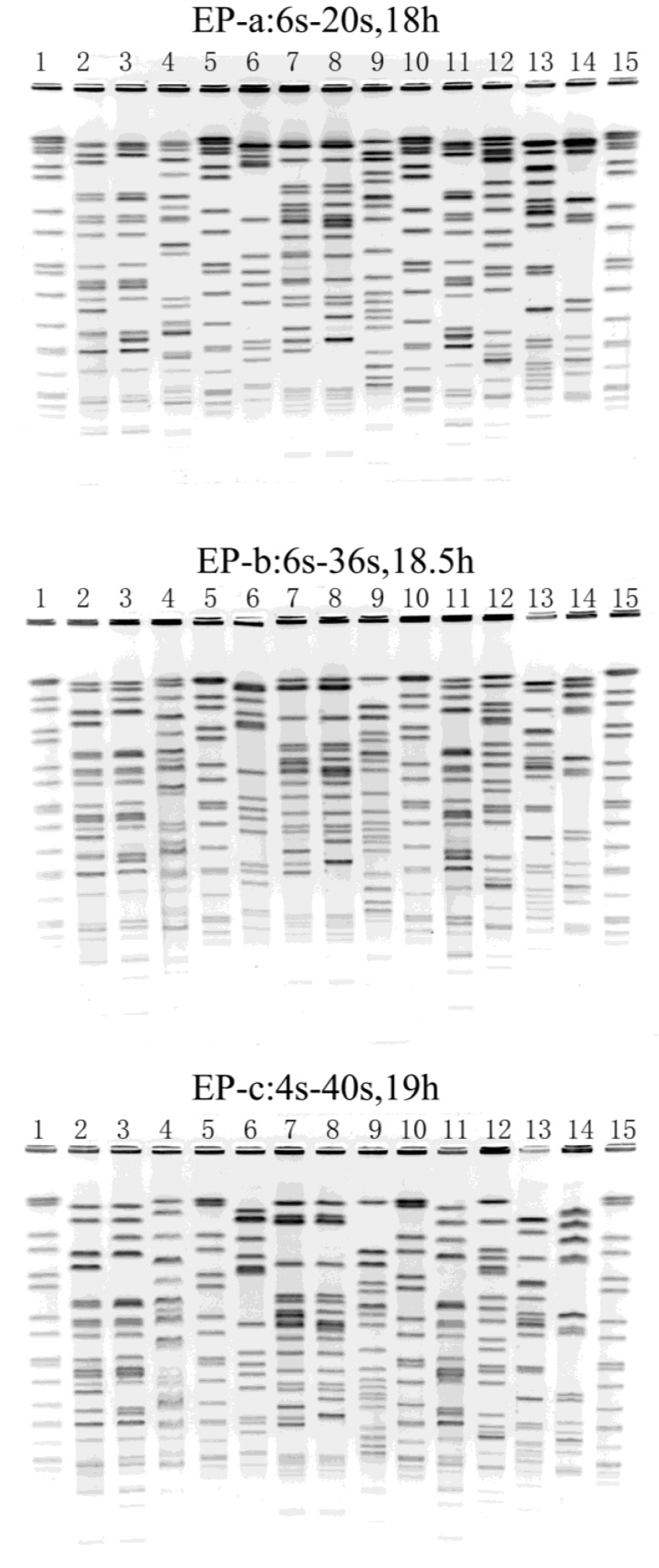
PFGE images obtained using three EPs with *Xba*I digestion. Lands 1, 5, 10, 15 were DNA size marker *Salmonella* serotype Braenderup H9812. Lands 2–4, 6–9, 11–14 were tested *K. pneumonia.*

For every enzyme, an EP could be recommended by the CHEF Mapper equipment, based on the sizes of restriction fragments. The EP recommended for *Xba*I digestion of *K. pneumoniae* was a switch time of 6 to 20 s for 18 h (EP-a). However, with this EP, the bands were not well distributed, and it was not sufficient to distinguish big fragments (Figure 1). We fine-tuned the EP to provide the best possible resolution, and two more EPs were obtained (EP-b and EP-c). With the EP-c (4 s to 40 s for 19 h), some small fragments were mot sufficient to distinguish (Figure 1). So EP-b (6 s to 36 s for 18.5 h) was selected as the optimal EP of *Xba*I digestion and used in further study.

Then we used *Xba*I digestion with EP-b (6s to 36 s for 18.5 h) to analyses 69 *K. pneumoniae* strains. PFGE with *Xba*I digestion divided the 69 strains of *K. pneumoniae* into 54 different pulsotypes and gave a *D-*value of 0.9902.

### 3.2. Selection of Another Enzyme

**Figure 2:**
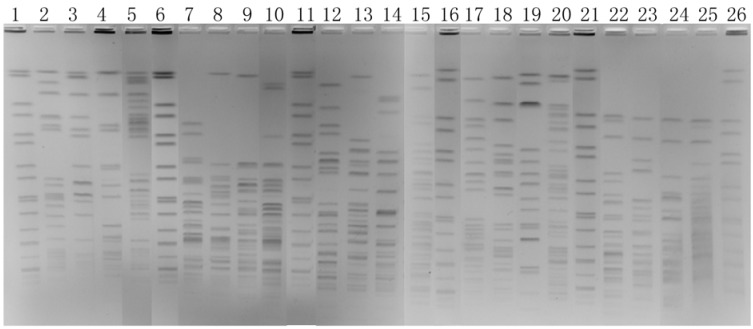
PFGE images of four *K. pneumoniae* isolates restricted with *Avr*II (lanes 2 to 5), *Pme*I (lanes 7 to 10), *Spe*I (lanes 12 to 15), *Xba*I (lanes 17 to 20) and *Swa*I (lanes 22 to 25). The size standard (H9812) was loaded in lanes 1, 6, 11, 16, and 21.

A theoretical enzyme selection using the DNASTAR 5.01 software was based on the complete genome sequence of *K. pneumoniae* subsp. *pneumoniae* MGH 78578 and included all satisfactory enzymes. *Avr*II, *Pme*I, *Spe*I, *Xba*I and *Swa*I digestion gave appropriate number of bands (10–50 bands) and were chosen as the candidate enzymes for the pilot study. We obtained four images using the five enzymes and found that besides *Xba*I, *Avr*II was gave images clear enough to meet our needs (Figure 2).

### 3.3. Typeability, Reproducibility and Discriminatory Power

PFGE with *Xba*I and *Avr*II digestions had the ability to type all *K. pneumoniae* strains and achieved the satisfactory typeability of 100%. Five isolates selected randomly were reanalyzed 3 times, and the patterns of the same isolate from different runs were defined to be indistinguishable, proving good reproducibility of the PFGE protocol optimized in this study (data not shown).

**Figure 3:**
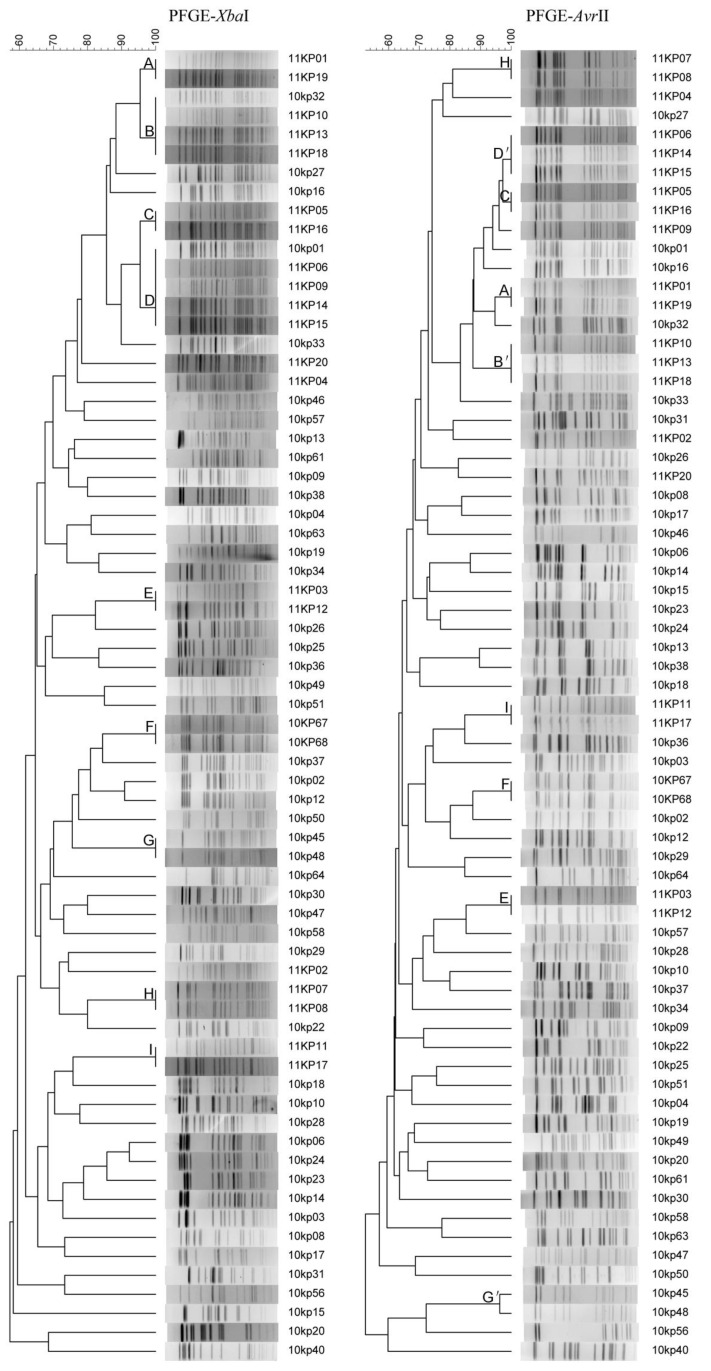
Clustering results of patterns obtained with *Xba*I- and *Avr*II-PFGE respectively. Charts are shown for 69 *K. pneumoniae* strains.

**Figure 4:**
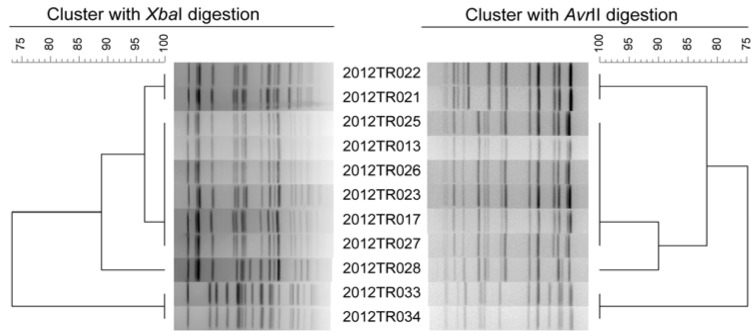
Clustering results of patterns obtained with *Xba*I- and *Avr*II-PFGE of 11 *K. pneumoniae* strains from one outbreak.

We analyzed the 69 strains with the EP of a switch time of 6 to 36 s for 18.5 h and could provide reasonable patterns. PFGE with *Avr*II digestion divided the 69 strains of *K. pneumoniae* into 59 different pulsotypes and gave a *D-*value of 0.9949, slightly higher than that of *Xba*I digestion (Figure 3). There were nine groups (Groups A to I in Figure 3) of strains could not be divided by *Xba*I digestion. Among them, groups A, C, E, F, H, I also could not be divided by *Avr*II digestion; however, other three groups (Groups B/B’, D/D’ and G/G’ in Figure 3) were divided by *Avr*II digestion, which showed higher discriminatory power compared to *Xba*I digestion. We then analyzed 11 isolates from one outbreak to evaluate the discriminatory power of PFGE method to divided outbreak strains (Figure 4).These 11 strains were isolated from a same ward during four days. *Xba*I- and *Avr*II-PFGE gave almost the same subtyping results. Both *Xba*I- and *Avr*II-PFGE divided 11 strains into four PFGE types and clustered six of 11 strains together with same patterns.

### 3.4. Concordance between PFGE and MLST Methods

**Figure 5:**
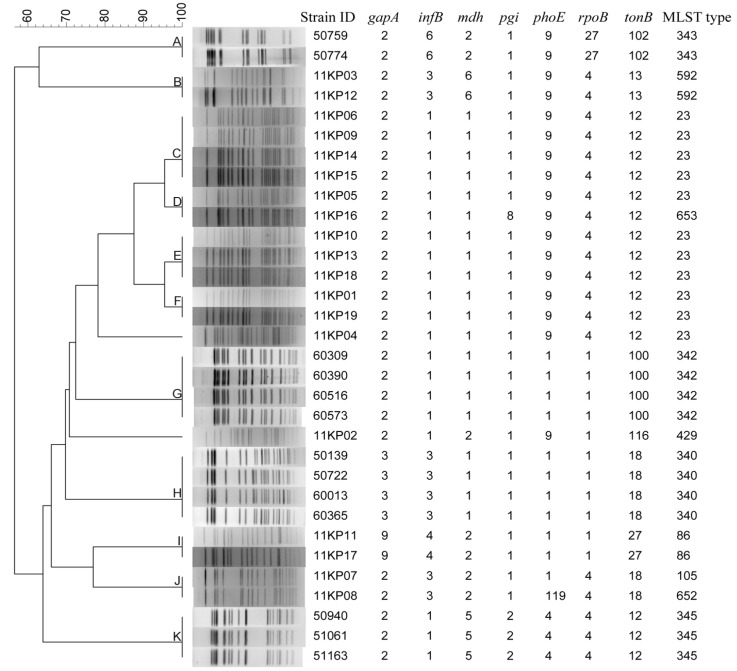
Clustering results of patterns obtained with *Xba*I digestion of 32 *K. pneumoniae* strains tested by multilocus sequence typing.

Two panels of *K. pneumoniae* isolates were used to evaluate the concordance between PFGE and MLST. The first panel contained 32 strains with no direct epidemiological relation (Figure 5).

PFGE distinguished the 32 strains into 13 patterns, whereas 11 types were obtained by MLST. Overall, the cluster results of PFGE and MLST have high concordance. The strains had same PFGE patterns also have same (Groups A, B, C, E, F, G, H, I and K in Figure 5) or similar (Groups D and J in Figure 5) MLST types. All the strains belonging to same MLST types had same PFGE patterns except that of ST23. The strains belonging to ST23 were divided into five different PFGE types (Groups C, D, E, F and strain 11KP04).

**Figure 6:**
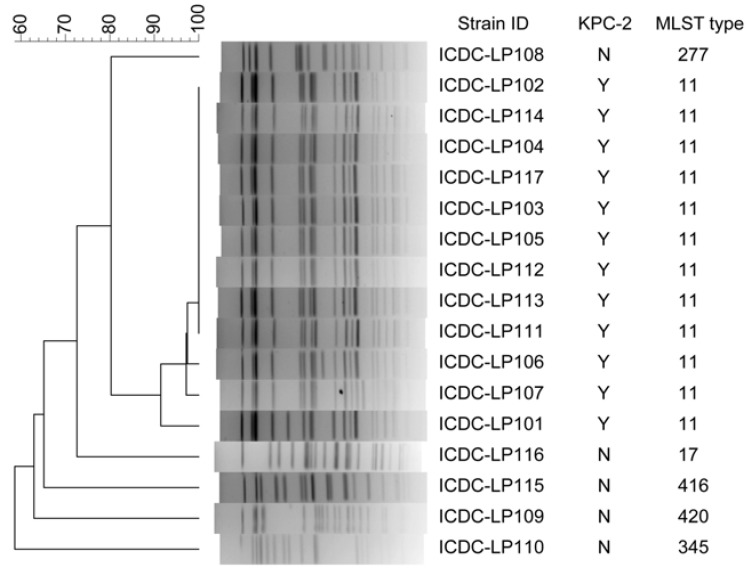
Comparing of PFGE (*Xba*I digestion) and MLST to subtype *K. pneumoniae* strains isolated during an outbreak period.

The second panel contained 17 *K. pneumoniae* isolated from a same ward in a hospital at a same time, showing parts of the isolates maybe have direct epidemiological relation (Figure 6). All the 12 *K. pneumoniae* carbapenemase 2-producing (KPC-2) *K. pneumoniae* strains belonged to ST-11 by MLST analysis. By PFGE, these 12 KPC-2 strains were divided into four different pulsetypes; nine of them had dominant pulsetype and other three pulsetypes had one to three bands different to the dominant one. The five non- KPC-2 strains had higher polymorphism when analyzed by both PFGE and MLST.

## 4. Discussion and Conclusions

PFGE has a high degree of reproducibility and unprecedented resolving power. With this method, databases can be established and the standardization of data communication between laboratories can be achieved. Therefore, PFGE is often considered as the “gold standard” of molecular typing methods. Standardization of protocols is crucial for the successful implementation of any molecular method as a practical epidemiologic tool.

Previous studies have shown several factors could influence PFGE results, including plug preparation, enzymatic digestion and electrophoresis [20,21,22]. We have optimized the cell concentration in plug preparation procedure, which influence the quality of patterns (data not shown). The concentration of bacterial cells is very important for the experiment. If the concentration is too low, the image will be too dim to be seen clearly. Conversely, the too high concentration will lead to high background of the PFGE patterns, partial digestion, and poor resolution. In this study, the cell suspensions with an optical turbidity near 4.0 units using a bioMérieux DENSIMAT worked best for PFGE of *K. pneumoniae*. The “ghost” and “phantom” bands were observed when the turbidity adjusted to 4.0 or higher. Additionally, different restriction enzymes produce different banding patterns and often different band numbers, which would influence the typing effect of PFGE [23]. The principle for the selection of a restriction enzyme is to find one that can produce clear patterns with uniformly distributed bands. Fewer bands reflect less information from the image of the bands, while too many bands reduce the separation of the bands where overlap occurs. Early reports of standardized PFGE protocols demonstrated that the combination of two restriction enzymes increased the discriminatory power of the method [11,24]. Thus, two enzymes were needed in PFGE for subtyping of *K. pneumoniae*. By means of software analysis and pilot study, *Xba*I and *Avr*II were chosen as the enzymes based on clear patterns, the appropriate number of bands, and the distribution of bands.

Several criteria have been proposed for evaluating the performance of typing systems including typeability, reproducibility, and most importantly, discriminatory power [25]. Discriminatory power of a method is the ability to distinguish between unrelated isolates, ideally assigning each to a different type. Discrimination indices and the similarity coefficients are the index usually used to compare discriminatory power. Restriction enzymes and EP used are the two factors influencing discriminatory power. In this study, we chose 69 *K. pneumoniae* strains of different origins to compare the discriminatory ability of *Xba*I- and *Avr*II-PFGE. These strains have much less epidemiological relation. This permits to use *D*-value to estimate the discriminatory power of the typing methods. Both *Xba*I- and *Avr*II-PFGE gave a *D*-value higher than 0.99. Therefore, this combination could be used as a typing method for *K. pneumoniae* with the generally accepted probability of 1% of type I errors (Struelens 1996). Because *Xba*I is cheaper than *Avr*II (30 times cheaper from New England Biolabs), so we recommend the use of the *Xba*I as the primary enzyme, and then *Avr*II used when further differentiation is needed.

In conclusion, this study aimed to optimize the PFGE procedure of *K. pneumoniae* and an appropriate protocol was developed. This protocol is anticipated to be used widely for *K. pneumoniae* genotyping for epidemiological surveys and in research.
